# Unusual Location of a Rare Tumor: Cutaneous Myxoma of the Scrotum

**DOI:** 10.7759/cureus.67911

**Published:** 2024-08-27

**Authors:** Keerthana Devi D, Priyathersini Nagarajan, Divya Dhanabal, Saravanan Sanniyasi

**Affiliations:** 1 Pathology, Sri Ramachandra Institute of Higher Education and Research, Chennai, IND; 2 General Surgery, Sri Ramachandra Institute of Higher Education and Research, Chennai, IND

**Keywords:** carney’s complex, angiomyxoma, myxoid, mesenchymal neoplasm, myxoma, scrotal swelling

## Abstract

Myxoma is a rare benign mesenchymal neoplasm. Scrotal myxomas can be either cutaneous or intramuscular. Muscular myxomas are usually found in cardiac muscles and skeletal muscles of the extremities. The most common locations of cutaneous myxomas are the trunk, lower extremities, and the head and neck. However, these lesions can rarely arise in the genital area (vulva, mons pubis, and scrotum). The clinical presentation of this lesion is nonspecific, and it is difficult to make a diagnosis before a biopsy and microscopic examination. Scrotal myxomas are infrequent and can cause definite diagnostic problems.

We report a case of a 64-year-old male who presented to the surgery outpatient department with a history of scrotal swelling for 20 years. The swelling was excised and sent for histopathological examination. Histopathological examination revealed features of scrotal cutaneous myxoma.

## Introduction

Myxoma is a rare gelatinous benign neoplasm resembling the umbilical cord of the fetus. Cutaneous myxoma [1] is otherwise known as superficial angiomyxoma. Cutaneous myxoma is slightly more common in males. The usual age group is middle-aged adults. As mentioned earlier, these lesions rarely arise in the genital areas (vulva, scrotum, and mons pubis). Clinically, they present as slow-growing, papulonodular or polypoidal cutaneous lesions which may be confused with a skin tag, papilloma, or neurofibroma. Ultrasonography of cutaneous myxoma [2] in the scrotum reveals a well-circumscribed, heterogeneously echogenic ovoid lesion. In Doppler mode, vascular flow signals can be seen in and surrounding the lesion.

Grossly, they are mostly well-circumscribed but few are poorly circumscribed. They mostly range in size from 1 to 5 cm and have a gelatinous cut surface. Microscopically at low magnification, it has a lobular appearance. The lesion is composed of extensive myxoid stroma with sparse spindle to stellate-shaped cells. Prominent vasculature which is focally arborizing can also be seen. Some tumors [3] have epithelial components like epithelial strands, basaloid buds, or epidermoid cysts. By immunohistochemistry, the lesional cells [4] consistently express CD 34 and rarely express S100 protein or cytokeratins.

## Case presentation

A 64-year-old male presented with a gradual onset of a painless scrotal swelling for 20 years. The swelling gradually increased in size. There was no history of discharge from the swelling, trauma to the scrotum, fever, or any other similar swellings elsewhere in the body. There was no significant past and family history.

On examination, a firm, nontender, mobile swelling of size 1 x 0.5 x 0.5 cm was noted over the scrotum. The skin over the swelling was folded and irregular, and focal areas showed short fingerlike projections; however, no ulceration or redness was noted. Bilateral testes and cord structures were normal. Abdomen and inguinal examinations were normal. All other systems were unremarkable. The clinical provisional diagnosis was scrotal papilloma. The patient underwent excision of the swelling under local anesthesia. The postoperative period was uneventful.

**Figure 1:**
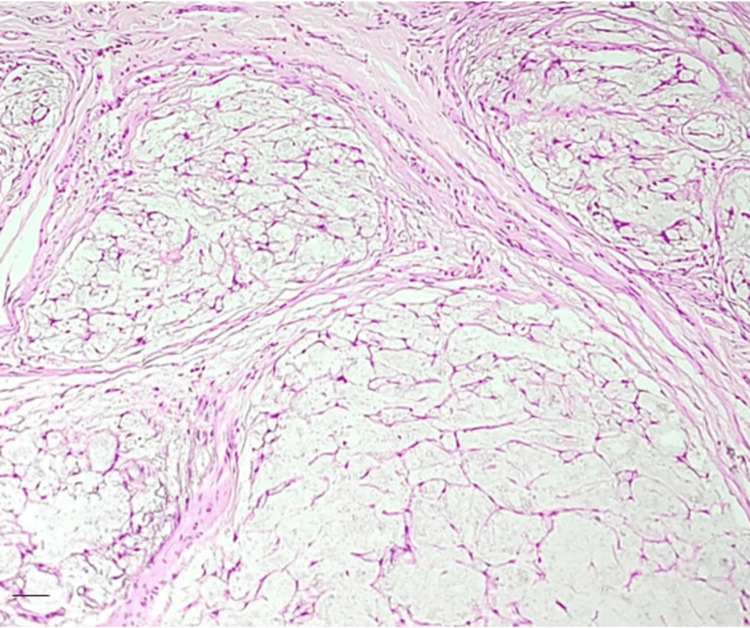
Multilobular architecture and extensive myxoid stroma Hematoxylin and eosin (H&E) stain: 100x magnification. Scale bar: 200 um

**Figure 2:**
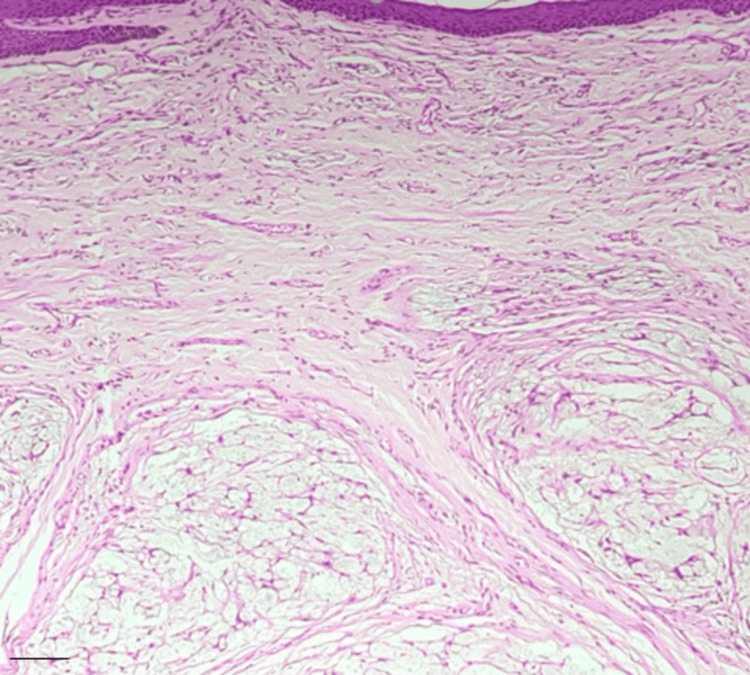
Dermal myxoid lesion Hematoxylin and eosin (H&E) stain: 100x magnification. Scale bar: 200 um

**Figure 3:**
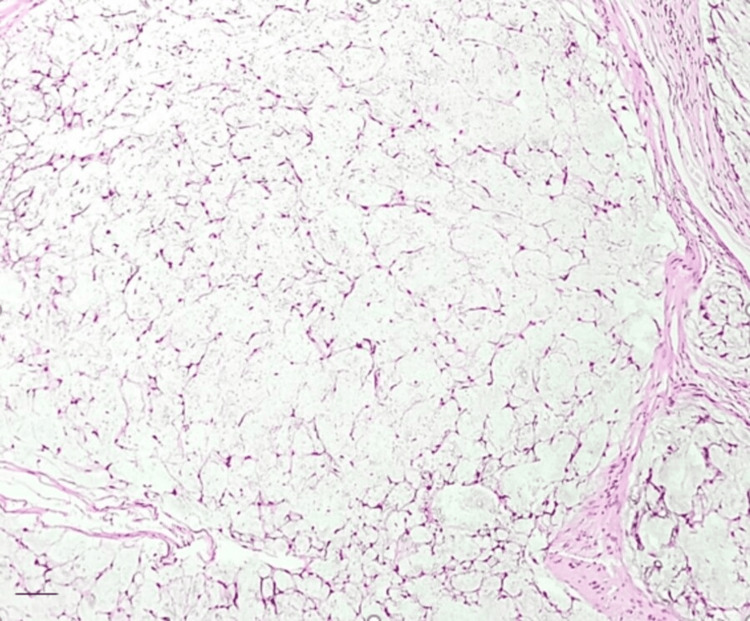
Scattered spindled to stellate-shaped cells deposited in the myxoid stroma. These cells have indistinct cell border and oval nuclei Hematoxylin and eosin (H&E) stain: 400x magnification. Scale bar: 40 um

Excised swelling was sent for histopathological examination. Grossly, the swelling was a skin-covered globular soft tissue mass measuring 1 cm in the greatest dimension. The cut surface of the mass was gray-white in color and soft in consistency. The entire tissue was embedded and studied microscopically. Microscopic examination revealed a dermal lesion with lobular architecture composed predominantly of myxoid matrix with few spindle-shaped cells. Occasional scattered thin-walled, small-sized blood vessels were also noted. There was no evidence of cytological atypia/atypical mitosis/necrosis. The histological features were highly suggestive of a benign mesenchymal neoplasm favoring cutaneous myxoma (Figures 1-3).

## Discussion

Differential diagnoses [1] of cutaneous myxoma are vast and include numerous benign and low-grade malignant myxoid neoplasms like deep angiomyxoma, focal cutaneous mucinosis, dermal nerve sheath myxoma, myxoid neurofibroma, myxoid liposarcoma, and myxofibrosarcoma.

Focal cutaneous mucinosis [1] does not have the lobular appearance which is present in cutaneous myxoma. Nerve sheath myxoma has a more pronounced lobular architecture and has plumper cells that stain positive for S100 immunohistochemistry. Myxoid neurofibroma has a characteristic collagen known as shredded carrot collagen, and the tumor cells have wavy nuclei, which is positive for S100. Myxoid liposarcoma is generally located deeper and larger in size than cutaneous myxomas. Histologically, it has a chicken wire or plexiform vasculature and scattered lipoblasts. Myxofibrosarcoma is characterized by a higher degree of nuclear atypia and hyperchromasia along with blood vessels that are curvilinear and lined by atypical tumor cells. Deep angiomyxoma [5] also known as aggressive angiomyxoma is much larger and involves deeper structures compared to the cutaneous myxoma. Aggressive angiomyxoma occurs usually in the genital region of women and males are rarely affected, whereas cutaneous myxoma occurs commonly in extragenital sites and the incidence is slightly more common in males. 

In 1988, Allen et al. [6] analyzed 28 cases of cutaneous myxoma. The mean age group was 39 years and the majority of them ranged in size from 1 cm to 5 cm. There were 12 female and 16 male patients. The most common location was the trunk (11 cases) and lower extremity (10 cases) followed by the head or neck (five cases) and upper extremity (four cases).

In 1999, Calonje et al. [7] analyzed 39 cases of cutaneous myxoma. In this case series, the lesion was more common in males. The age of presentation ranged from zero to 82 years, and the size ranged from 1 cm to 5 cm. The most common location was trunk (17 cases) followed by head and neck (14 cases) and lower limbs (seven cases).

According to the literature [8], only seven cases of cutaneous myxomas have been reported to arise on the scrotum. Among the seven cases, the most common age group was 3rd to 5th decade, and the mean size of the lesion was approximately 3 cm.

In any case of cutaneous myxoma [9] presenting at early adulthood, the possibility of Carney’s complex should be kept in mind. Carney’s complex is a rare autosomal dominant disorder which manifests as multiple cutaneous myxomas, lentigines, cardiac myxoma, and endocrine hyperactivity. A meticulous follow-up of a patient with cutaneous myxoma is essential, as it can be the first and only manifestation of Carney’s complex.

Cutaneous myxomas [10] do not exhibit metastatic potential. However, they [11] have a high rate of local recurrence between 20% and 40%. Hence, complete surgical excision is very essential. Despite having a good overall prognosis, without deeper structure invasion, these patients must be followed up for long term.

## Conclusions

Although an uncommon diagnosis, myxoma should be considered as a differential diagnosis of scrotal tumors. Scrotal myxomas usually occur in 3rd to 5th decades of life. Since cutaneous myxomas have varied clinical presentations, histopathological examination is necessary for the diagnosis. Morphological findings are sufficient for making the diagnosis. Complete excision of the lesion is the preferred treatment. Since cutaneous myxomas have high chances of local recurrence, a close follow-up of the patient is necessary. 
